# *Leonotis ocymifolia* (Burm.f.) Iwarsson aerial parts aqueous extract mitigates cisplatin-induced nephrotoxicity via attenuation of inflammation, and DNA damage

**DOI:** 10.3389/fphar.2023.1221486

**Published:** 2023-08-01

**Authors:** Afoua Mufti, Anouar Feriani, Wafae Ouchari, Yasmine M. Mandour, Nizar Tlili, Mohammed Auwal Ibrahim, Mona F. Mahmoud, Mansour Sobeh

**Affiliations:** ^1^ Laboratory of Biotechnology and Biomonitoring of the Environment and Oasis Ecosystems, Faculty of Sciences of Gafsa, University of Gafsa, Gafsa, Tunisia; ^2^ AgroBioSciences Program, College for Sustainable Agriculture and Environmental Science, Mohammed VI Polytechnic University, Ben Guerir, Morocco; ^3^ School of Life and Medical Sciences, University of Hertfordshire Hosted By Global Academic Foundation, Cairo, Egypt; ^4^ Institut Supérieur des Sciences et Technologies de L’Environnement, Université de Carthage, Carthage, Tunisia; ^5^ Department of Biochemistry, Ahmadu Bello University, Zaria, Nigeria; ^6^ Department of Pharmacology and Toxicology, Faculty of Pharmacy, Zagazig University, Zagazig, Egypt

**Keywords:** *Leonotis ocymifolia*, nephroprotective, oxidative stress, antioxidant, DNA fragmentation

## Abstract

Herein, we explored the protective effect of *Leonotis ocymifolia* (Burm.f.) Iwarsson aerial parts extract (LO) against cisplatin (CP)-induced nephrotoxicity in rats and profiled their phytocontents. A total of 31 compounds belonging to organic and phenolic acids and their glycosides as well as flavonoids and their *O*- and *C*-glycosides were identified through LC-MS/MS. The DPPH and FRAP assays revealed that the extract had powerful antioxidant properties. The *in vivo* results demonstrated that administering LO extract for 30 days (40 and 80 mg/kg b. w.) significantly improved the altered renal injury markers via reducing creatinine (high dose only) and uric acid levels compared to the Cp-group. The deleterious action of cisplatin on renal oxidative stress markers (GSH, MDA, SOD, and CAT) were also mitigated by LO-pretreatment. The reduction of the inflammatory marker (IL-6), and inhibition of DNA fragmentation, highlighted the prophylactic action of LO in kidney tissue. Molecular docking followed by a 100 ns molecular dynamic simulation analyses revealed that, amongst the 31 identified compounds in LO, chlorogenic and caffeoylmalic acids had the most stable binding to IL-6. The nephroprotective effects were further confirmed by histopathological observations, which showed improvement in ultrastructural changes induced by cisplatin. The observed findings reinforce the conclusion that *L. ocymifolia* extract exerts nephroprotective properties, which could be related to its antioxidant and anti-inflammatory activities. Further studies are required to determine the therapeutic doses and the proper administration time.

## Introduction

The kidneys play a pivotal role in the filtration and purification of blood plasma, effectively eliminating metabolic waste products such as urea, creatinine, uric acid, and urates through the intricate processes of filtration, tubular secretion, and re-absorption of various substances. Additionally, the kidneys maintain the appropriate concentrations of water and electrolytes, thus aiding in the regulation of blood volume and composition and glomerular filtration (Saatkamp et al., 2016). Renal disease currently stands as a significant public health concern, affecting millions of people in the world (Crews et al., 2019).

Cisplatin, also known as cis-dichlorodiammine platinum (II), is one of the most potent chemotherapeutic drugs used in the treatment of cancer. However, nephrotoxicity, myelosuppression, hypersensitive responses, ototoxicity, neurotoxicity, and bone marrow suppression have limited its application (Yassir et al., 2022). The hallmark of cisplatin nephrotoxicity is the significant death of renal proximal tubular (RPT) cells, by both necrosis and apoptosis (Cao et al., 2018). Cisplatin exerts cytotoxic effects through various mechanisms, including the inhibition of protein synthesis, mitochondrial injury, and DNA damage, which ultimately activate programmed cell death pathways in both tumor and renal tubular cells (Santos et al., 2007).

Kidney eliminates cisplatin from the body by glomerular filtration and tubular secretion (Yao et al., 2007). It is well-known that the overproduction of free radicals, that leads to oxidative stress, is implicated as important mediators of many diseases, such as acute kidney injury (Baek et al., 2003). It has been reported that cisplatin generates reactive oxygen species (ROS) and declines the activity of antioxidant enzymes leading to enhanced lipid peroxidation in renal tissues (İşeri et al., 2007). So far, there are no medical treatments that are effective in either preventing or curing nephrotoxicity caused by cisplatin. The only available drug for protection against cisplatin induced nephrotoxicity, is amifostine, but it is not commonly used in clinical practice because of its adverse effects especially hypotension, nausea and vomiting (Zhang et al., 2023). Therefore, it is essential to develop a medication that is both safe and effective for preventing kidney damage caused by cisplatin. A large number of naturally occurring compounds found in plants possess antioxidant and anti-inflammatory properties, which can provide a wide range of health benefits, including protection against oxidative stress by restoring the antioxidant enzymes levels, this refers to a strategy that holds significant interest for chemotherapy (Fang et al., 2021; Yassir et al., 2022).

*Leonotis* is a small genus belonging to the Lamiaceae (mint) family. The medicinal qualities of various *Leonotis* species are renowned. *Leonotis ocymifolia* (Burm.f.) Iwarsson, commonly known as minaret flower or KlipDagga, is a roughly hairy shrub, widespread throughout southern and eastern Africa (Vagionas et al., 2007). *Leonotis ocymifolia* has several reputed traditional medicinal uses. It is employed for the treatment of headaches, ulcers of the neck and swelling and serves as an ascaricide. The roots and the flowers are used against gout, leishmaniasis and cancer (Habtemariam et al., 1994; Eguale et al., 2011). Moreover, numerous pharmacological activities of *L. ocymifolia* have been investigated. The aqueous and the hydro-alcoholic extracts of its aerial parts, for instance, demonstrated some degree of anthelmintic activities against egg and larvae of *Haemonchus contortus* (Eguale et al., 2011). The essential oil of *L. ocymifolia* displayed a noteworthy antimicrobial activity against *Staphylococcus epidermidis* and *Staphylococcus aureus* (Vagionas et al., 2007). Three compounds were identified and isolated from the aerial parts using H and ^13^C NMR spectra, namely, leonotin, leonotinin and nepetaefolin (Hussein et al., 2003).

In this work, we identified the chemical composition of *L. ocymifolia* aerial parts extract using LC-MS/MS. Then, we studied its protective activity against cisplatin induced nephrotoxicity in rats *via* measuring several biochemical markers, histopathological examination, and investigation of DNA fragmentation. Finally, we performed molecular modeling and molecular dynamics studies of the annotated metabolites on IL-6, an important proinflammatory cytokine implicated in cisplatin induced nephrotoxicity.

## Materials and methods

### Plant material, extraction, LC-MS and *in vitro* assays

The air-dried aerial parts of *L. ocymifolia* (Burm.f.) Iwarsson were finely powdered, then subjected to ultrasound-assisted extraction (UAE) (50 g x 750 mL) using Sonics Vibra-Cell (Sonics and Materials, Inc., CT 06470–1614 United States) following the extraction parameters: 20 Hz, 5°C, amplification of the sound wave 30%, for 15 min and a pulse of 10 s. The filtered extract was centrifuged (6,000 rpm, 7 min) and evaporated (Buchi rotavapor^®^ R-300, Flawil, Switzerland) yielding a fine dried extract (7.1 g). HPLC-PDA-MS/MS was performed as described (Tawfeek et al., 2023). Total phenolic content (TPC), total flavonoids content (TFC), DPPH, and FRAP assays were performed according to Ghareeb et al. (Ghareeb et al., 2018).

## *In vivo* study

### Animals

Wistar rats (Male, similar age, around 160 g) were obtained from the Central Pharmacy, Tunisia. The rats were housed at the laboratory cages (Faculty of Sciences, Gafsa, Tunisia) under controlled conditions (temperature: 24°C ± 2°C, 55% ± 5% relative humidity, 12 h light/dark cycle, fed on a standard chow diet and water *ad libitum*).

### Induction of nephrotoxicity and experimental design

Cisplatin was dissolved in saline and injected to animals intraperitoneally at the dose of 13 mg/kg body weight (b.w.) to induce nephrotoxicity as per previous studies (Domitrović et al., 2013). Forty animals were divided into 5 groups (*n* = 8). One of the groups was regarded as control and received oral treatment with vehicle control (saline) while the remaining four groups received cisplatin (CP, 13 mg/kg b. w.), a single dose (IP) on day 30. Among these groups, two were selected and given an oral treatment with LO extract (40 mg/kg b. w.) and (80 mg/kg b. w.) respectively for 30 days while one group received an oral treatment with the reference drug, cystone (100 mg/kg b. w.) for 30 days. The last group of cisplatin induced animals was left untreated.

### Biochemical assays

Animals of each group were sacrificed at the end of the experiments. Blood samples were collected, and the plasma was obtained after centrifugation at 2000 *g* for 15 min. The plasma was maintained at −20°C to evaluate numerous biochemical parameters, including markers of renal injury (urea, uric acid, and creatinine) using colorimetric kits (Sigma-Aldrich) according to the manufacturer’s protocol. The ELISA assay for IL-6 in whole-kidney homogenates was performed using diagnostic kits from Biomaghreb (Tunisia) and according to the manufacturer’s instruction. The levels of electrolytes including Na^+^, and K^+^, in plasma were measured with an ionogramme analyzer (EasyLyte Plus, Medica, France).

The kidney tissues were put on ice then washed with normal saline. Then after, it was homogenized in potassium phosphate buffer (0.1 M, pH 7.4). The resultant mix was centrifugated for 15 min at 12,000 rpm (4°C) to recover the supernatant. The latter was utilized to determine malondialdehyde (MDA) levels through thiobarbituric acid reaction and was presented as nmol concentration of MDA content per mg of protein (Buege and Aust, 1978). The total activity of SOD in the supernatant was evaluated *via* the inhibition of pyrogallol autoxidation catalyzed by the superoxide radical as previously described (Marklund and Marklund, 1974) and expressed as nM/mg protein. The GSH levels in the tissue homogenate was determined as described by Sedlak and Lindsay (Sedlak and Lindsay, 1968), with few modifications whilst the activity of catalase (CAT) was evaluated utilizing the method described by Aebi (Aebi, 1984) with minor modifications. The results were expressed as unit catalase activity per mg protein.

### Histopathological examination and DNA fragmentation assay

A portion of the kidney samples was immediately fixed in 10% buffered formalin phosphate solution. It was then embedded in paraffin, cut into 5 μm sections and stained using Hematoxylin and Eosin (H&E) and examined by light microscope for histological changes. The DNA in the renal tissue was extracted by phenol-chloroform-isoamyl alcohol method as previously described (Chtourou et al., 2015).

### Molecular docking

To gain insight into the interaction between some of the identified compounds in the LO extract and the inflammatory markers, molecular docking and dynamic simulation analyses were conducted. The crystal structure of IL-6 bound to its receptor gp 80 and an epitope antibody (PDB code: 5FUC) (Adams et al., 2017) was downloaded from Protein Data Bank. The structure preparation wizard in MOE (version 2019.01) was used to prepare the protein and saved as mol2 files. The 3D structure of the compounds was built and minimized using the MMFF94x force field in MOE using a gradient of 0.0001 kcal/mol Å. The binding site residues of gp 80 were used to define the binding site. GOLD (version 2022.2) (Jones et al., 1995; Jones et al., 1997) was used to conduct the docking experiments using Goldscore scoring function. All figures were prepared using PyMol (Schrödinger and Inc, 2015).

### Molecular dynamics simulation

MD simulations were carried out using the PMEMD. cuda code of the AMBER Molecular Dynamics package (Case et al., 2021) following the same previously described protocol of minimization, heating, density equilibration and a 100 ns production (Mandour et al., 2022). The trajectories were analyzed using CPPTraj (Roe and Cheatham, 2013). Plots and visual inspection of the trajectories were done using XMgrace (Turner, 2005) and VMD (Humphrey et al., 1996), respectively. The last 50 ns of the MD trajectory of each complex was used to compute the binding free energy using the MM-GBSA method (Genheden and Ryde, 2015) using the MMPBSA. py script (Miller et al., 2012) included in the AmberTools as previously described (Mandour et al., 2022).

### Statistical analysis

The data were expressed as mean ± Standard Error of Mean (mean ± SEM). The statistical difference among treatments was determined *via* One-Way Analysis of Variance (ANOVA), followed by Tukey *post hoc* test for multiple comparisons. The variance between the experimental groups was considered significant at *p* < 0.05. GraphPad Prism 6 (GraphPad Prism Software, San Diego, CA) was used to carry out all analyses.

## Results

### Phytoconstituents of *Leonotis ocymifolia* using LC-MS

**FIGURE 1:**
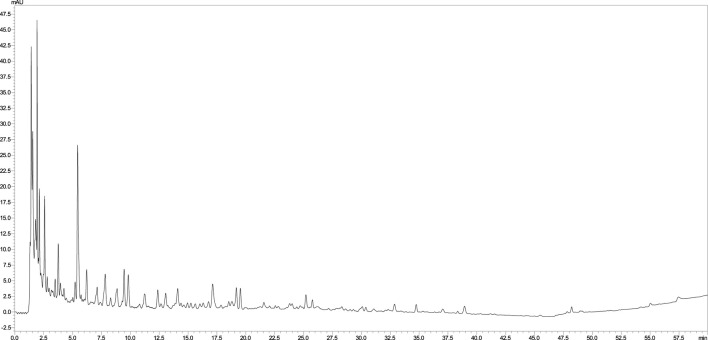
LC chromatogram of *Leonotis ocymifolia* aerial parts extract.

*Leonotis ocymifolia* aerial parts extract analysis furnished 31 compounds, *via* LC-MS/MS, belonging to organic acids and their dimers, phenolic acids and their glycosides as well as flavonoids and their *O-* and *C-*glucoside (Table 1; Figure 1). For organic acids, 4 signals were detected including citric acid, and malic acid along with its dimer and glucoside (Table 1). Several phenolic acids were also identified. For instance, two peaks showed [M-H]^-^ at *m/z* 345, and 355 and a fragment ion at 193 and were tentatively annotated as ferulic acid gallate and feruloyl glucose, respectively. Two other signals furnished 371 and 329 with a fragment ion at 209 and were tentatively assigned to caffeoylglucaric acid and hydroxybenzoyl glucaric acid, (Table 1). Other phenolic acids were also found including rosmarinic, protocatechuic, chlorogenic and caffeic acids (Table 1). Regarding flavonoids, 5 peaks were present including apigenin di-*C*-glucoside, di and tri glycosides of isorhamnetin and the aglycone, quercetin, (Table 1).

**TABLE 1:** Annotated compounds from *Leonotis ocymifolia* aerial parts extract.

No.	Rt	[M-H]^-^	MS/MS	Tentatively annotated compounds
1	1.45	191	111	Citric acid
2	1.64	133	115	Malic acid
3	2.05	249	133	Dimalic acid
4	3.37	295	133	Malic acid glucoside
5	3.74	331	169	Galloyl glucose
6	3.92	371	209	Caffeoylglucaric acid
7	4.88	315	153	Protocatechuic acid glucoside
8	5.06	331	153	Hydroxycaffeoyl protocatechuic acid
9	5.48	329	209	Hydroxybenzoyl glucaric acid
10	6.02	299	137	Hydroxybenzoic acid glucoside
11	6.20	371	209	Caffeoylglucaric acid isomer
12	6.21	153	109	Protocatechuic acid
13	7.04	447	153	Protocatechuic acid pentosyl-glucoside
14	9.33	299	137	Hydroxybenzoic acid glucoside
15	10.05	153	109	Dihydroxybenzoic acid
16	11.13	353	191	Chlorogenic acid
17	11.19	341	179	Caffeoyl glucose
18	11.31	325	163	Coumaric acid glucoside
19	11.92	345	193	Ferulic acid gallate
20	13.05	179	135	Caffeic acid
21	13.83	355	193	Feruloyl glucose
22	14.07	253	137	Hydroxybenzoic acid malate
23	15.64	287	183	Benzoyl methyl gallate
24	17.02	295	135	Caffeoyl malate
25	18.95	563	353	Apigenin *C*-pentosyl glucoside
26	21.83	295	133	Caffeoylmalic acid
27	24.95	755	315	Isorhamnetin rhamnosyl-pentosyl-glucoside
28	25.68	623	315	Isorhamnetin rhamnosyl-glucoside
29	31.16	359	179	Rosmarinic acid
30	34.20	461	315	Isorhamnetin glucoside
31	39.84	301	151	Quercetin

### *In vitro* assays

As expected, the *L. ocymifolia* aerial parts extract displayed marked antioxidant properties in DPPH and FRAP assays along with considerable amount of total phenolics (Table 2).

**TABLE 2:** *In vitro* results of *Leonotis ocymifolia* aerial parts extract.

Extract	DPPH	FRAP	TPC	TFC
	IC_50_, µg/mL	mM of FeSO_4_/g extract	mg GA/g extract	mg QE/g extract)
Extract	242.30 ± 8.60	22.62 ± 2.71	49.61 ± 1.64	1.17 ± 0.37
Ascorbic acid	53.65 ± 2.94	-	-	-
Quercetin	-	37.30 ± 2.29	-	-

### *In vivo* results

#### *Leonotis ocymifolia* modulates cisplatin induced renal dysfunction

**FIGURE 2:**
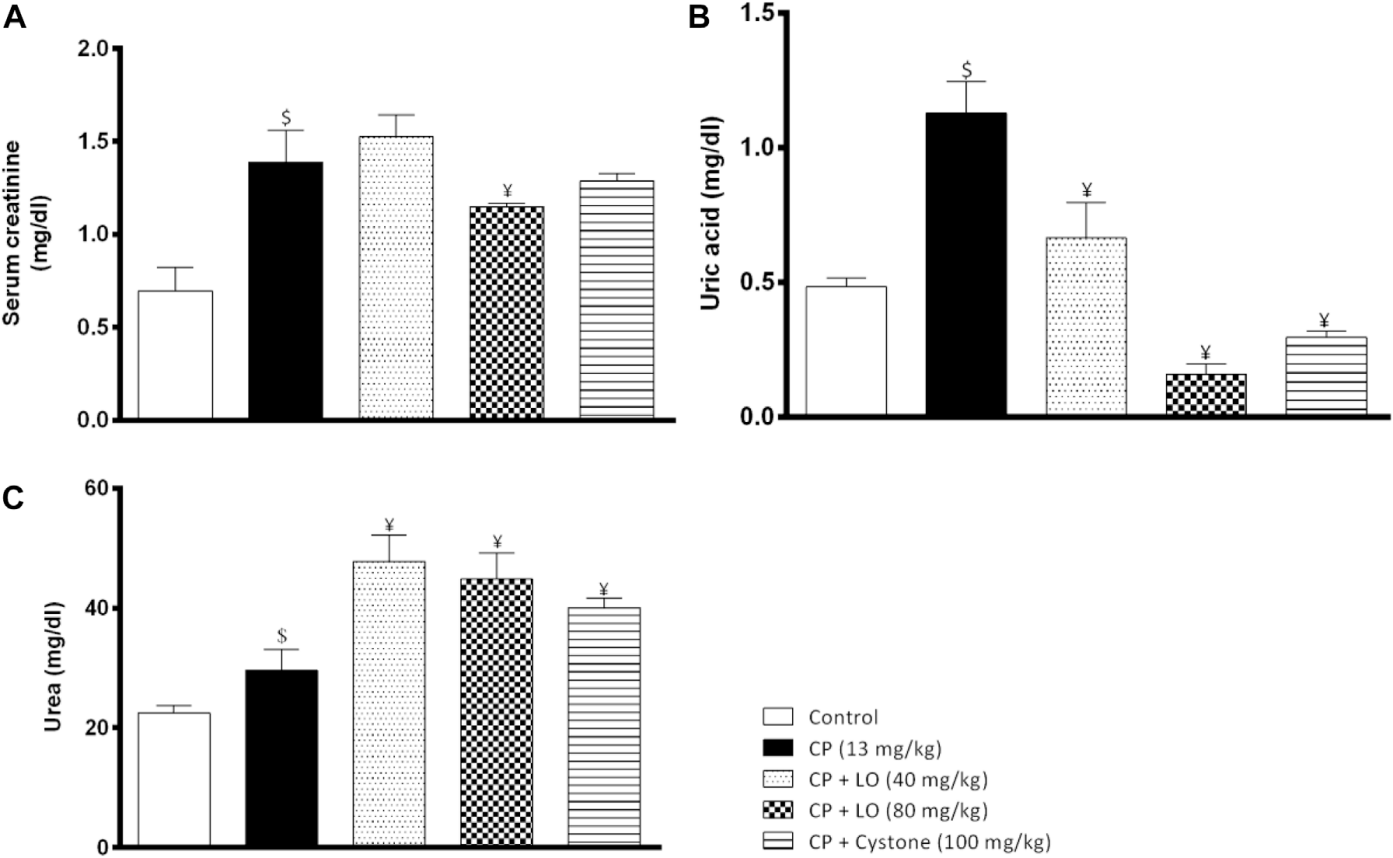
*Leonotis ocymifolia* modulates cisplatin induced renal dysfunction. Bar charts demonstrating the renal function levels following 30 days pretreatment with *Leonotis ocymifolia* extract (LO, 40 and 80 mg/kg, PO) and cystone as reference drug (100 mg/kg) followed by a single dose cisplatin (CP, 13 mg/kg, IP) **(A)** Serum creatinine **(B)** Serum uric acid level and **(C)** Serum urea. ^$,¥^ *p* < 0.05 vs. control, and cisplatin, respectively.

In the current investigation, results showed that the treatment with cisplatin significantly elevated serum levels of creatinine (Figure 2A), uric acid (Figure 2B) and urea (Figure 2C), indicating impaired renal functions and nephrotoxicity. The extract, dose dependently, decreased uric acid levels compared to cisplatin alone while surprisingly, it elevated the serum urea levels. Moreover, only the high dose of the extract decreased the elevated serum creatinine levels while neither the low dose nor cystone produced significant effect on serum creatinine level (*p* > 0.05).

#### *Leonotis ocymifolia* ameliorates cisplatin induced electrolyte abnormalities

**FIGURE 3:**
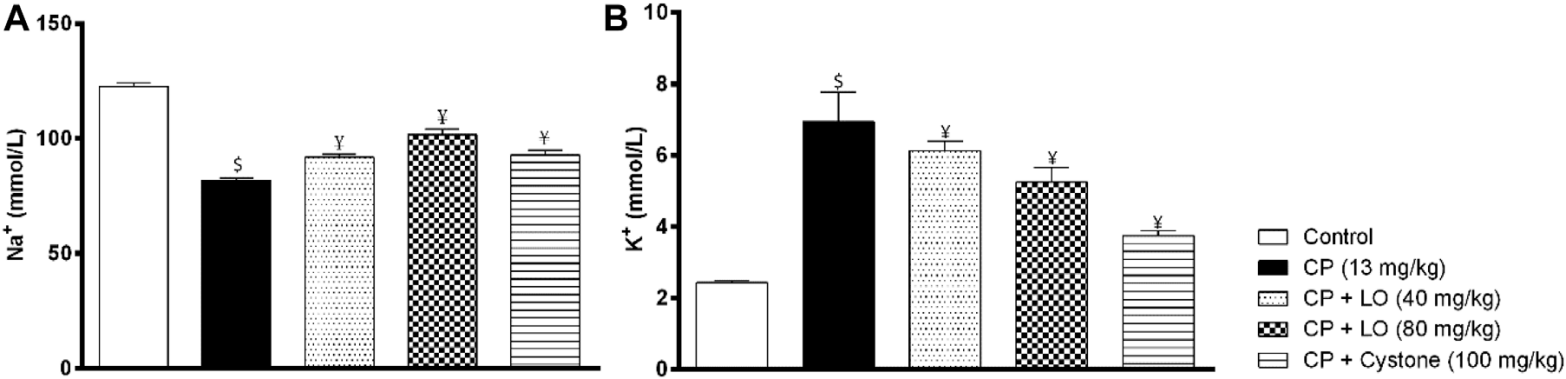
*Leonotis ocymifolia* ameliorates cisplatin induced electrolyte abnormalities. Bar chart demonstrating serum sodium levels **(A)** and plasma potassium levels **(B)** following 30 days pretreatment with *Leonotis ocymifolia* (LO, 40 and 80 mg/kg, PO) and cystone as reference drug (100 mg/kg) followed by single dose cisplatin (CP, 13 mg/kg, IP). ^$,¥^ *p* < 0.05 vs. control and cisplatin, respectively.

Results showed that LO extract, at both doses, restored decreased serum sodium level compared with cisplatin group (Figure 3A). On the other hand, the current investigation revealed that cisplatin produced hyperkalemia that was reversed by the extract (Figure 3B). The effect of the two dose levels of LO on serum potassium level was less potent than cystone (*p* > 0.05).

#### *Leonotis ocymifolia* (LO) modulates renal oxidative stress

**FIGURE 4:**
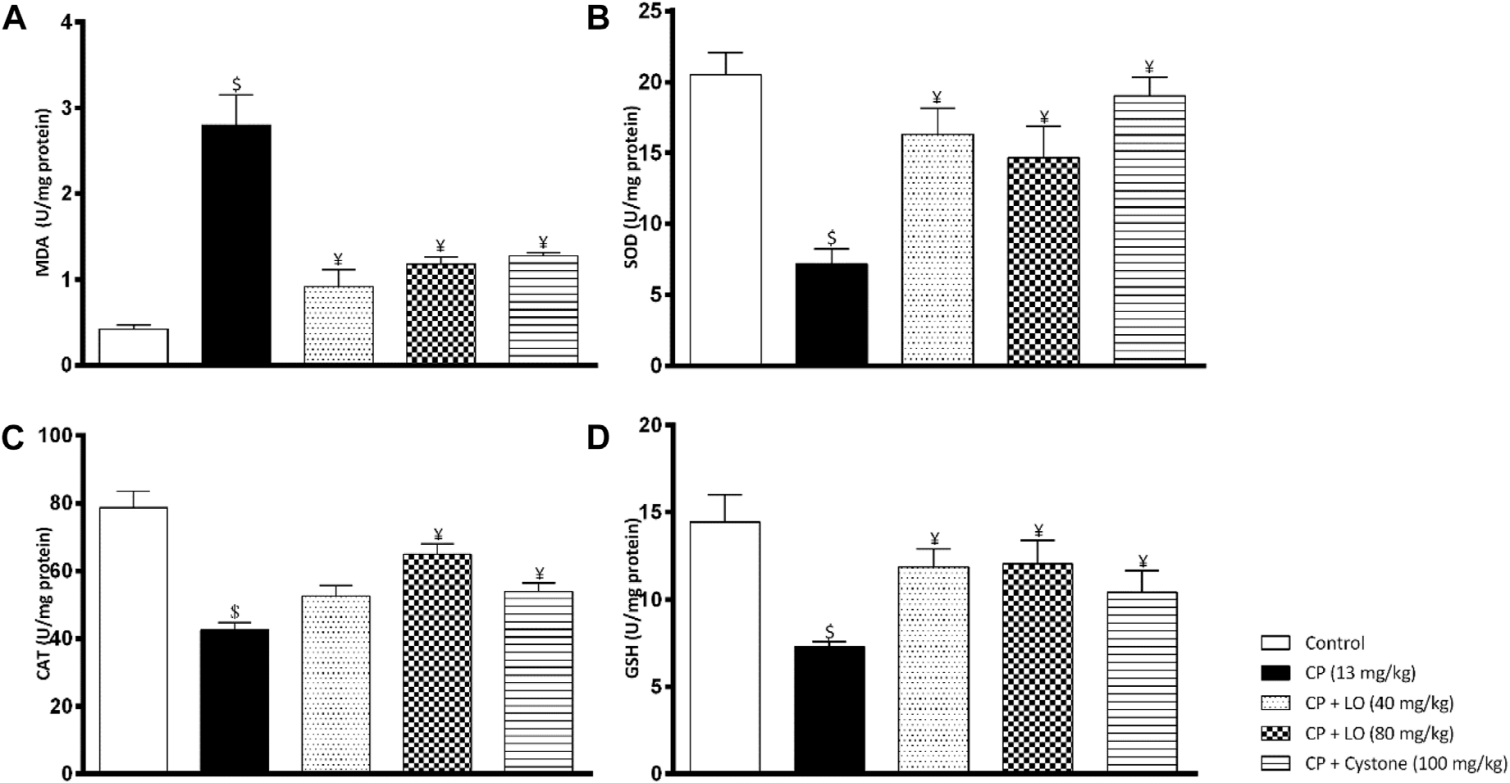
*Leonotis ocymifolia* (LO) modulates renal oxidative stress. Bar charts demonstrating renal lipid peroxidation product (malondialdehyde, MDA) level **(A)**, superoxide dismutase (SOD) content **(B)**, renal catalase (CAT) level **(C)** and renal reduced glutathione (GSH) content **(D)** following 30 days pretreatment with *Leonotis ocymifolia* (LO, 40 and 80 mg/kg, PO) and cystone as reference drug (100 mg/kg) followed by single dose cisplatin (CP, 13 mg/kg, IP). ^$,¥^ *p* < 0.05, vs. control, and cisplatin, respectively.

To determine the mechanism of nephroprotective effects of LO extract, we measured renal oxidative stress markers following cisplatin administration. The antioxidant capacity of LO detected using *in vitro* tests was confirmed by the *in vivo* results and showed that LO at both dose levels ameliorated cisplatin induced renal oxidative stress comparable to the reference drug, cystone. The extract suppressed lipid peroxidation in renal tissues as manifested by decreased lipid peroxidation product, MDA (Figure 4A). It also increased the activities of the enzymatic antioxidants such as SOD and catalase (Figure 4B, C). Furthermore, it elevated the level of the non-enzymatic antioxidants such as GSH compared with cisplatin group (Figure 4D).

#### *Leonotis ocymifolia* (LO) mitigates renal inflammation

**FIGURE 5:**
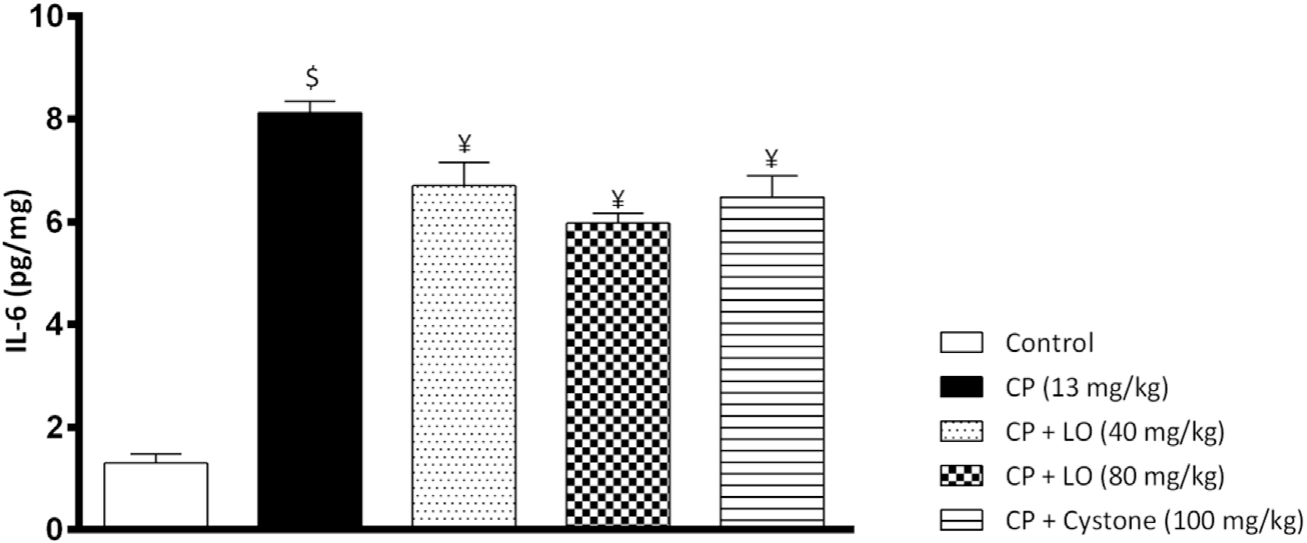
*Leonotis ocymifolia* (LO) mitigates renal inflammation. Bar chart demonstrating renal inflammation marker, interleukin-6 (IL-6) level following 30 days pretreatment with *Leonotis ocymifolia* (LO, 40 and 80 mg/kg, PO) and cystone as reference drug (100 mg/kg) followed by single dose cisplatin (CP, 13 mg/kg, IP). ^$,¥^ *p* < 0.05 vs. control, and cisplatin, respectively.

To investigate the effect of LO extract on renal inflammation, we measured one of the most important pro-inflammatory cytokines, IL-6. In the current study, cisplatin induced a significant increase in renal IL-6 compared to control group (Figure 5). Further, the present study found that LO extract dose dependently reduced renal IL-6 and exerted comparable or even better effects than cystone (Figure 5).

#### Molecular docking

In an endeavor to elucidate the observed nephroprotective effect of the extract, 9 of the identified compounds were docked to the crystal structure of IL-6 (PDB code: 5FUC) (Supplementary Table S1). The binding site residues of IL-6: Cys73, Phe74, Gln75, Phe78, Ser176, Leu178, Arg179, Ala180, Arg182 and Gln183 were used to define the docking site. The binding modes of the top-ranked compounds, 2-*O*-caffeoylglucaric acid, caffeoylmalic acid, rosmarinic acid and chlorogenic acid, showed promising binding modes, interacting with key residues in the structure of IL-6 that are also involved in the interaction of this cytokine with its receptor IL-6R or gp 80 (Kalai et al., 1997). To further confirm the binding of these compounds to IL-6, the stabilities of their binding modes were further evaluated by MD simulations.

**FIGURE 6:**
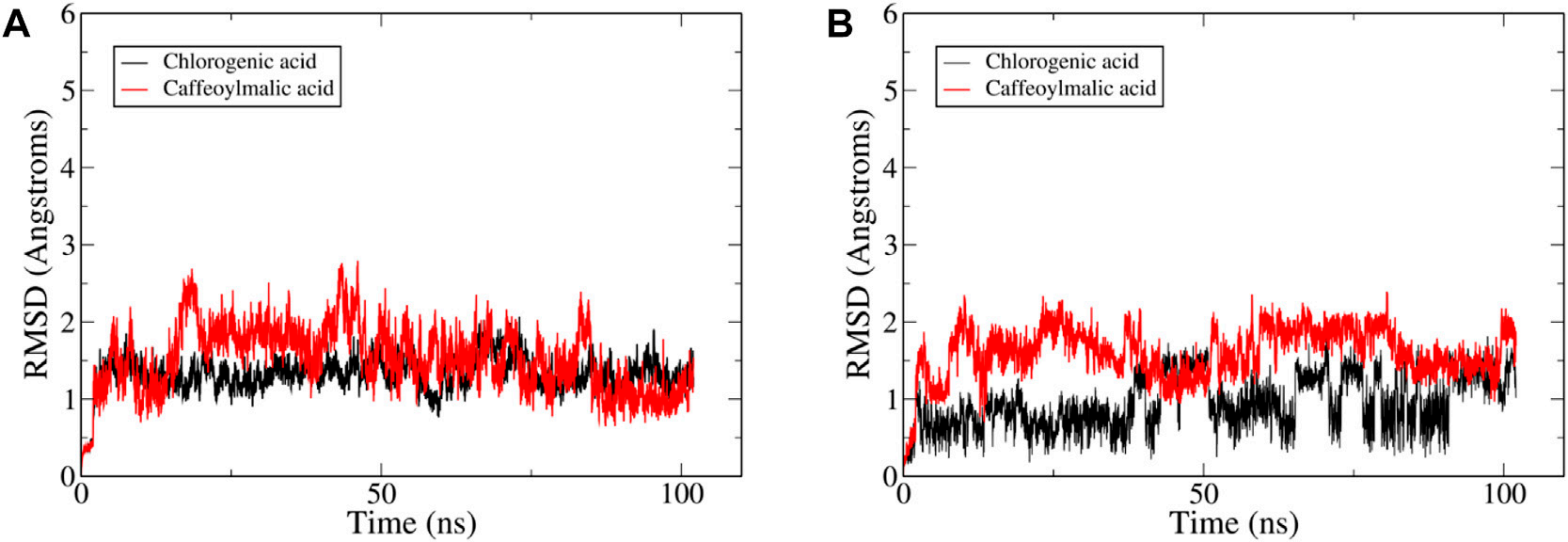
Plots of root-mean-square deviations (RMSD) of the entire trajectory with frames sampled every 20 ps? The plot depicts RMSD values based on **(A)** protein backbone atoms and **(B)** ligand heavy between the trajectory frames and the starting geometry for IL-6 complexed with compound—(red).

The reliability of the obtained docked poses was evaluated by running a 100 ns MD simulation for IL-6 with each one of these four compounds using AMBER software package (Case et al., 2021). Visual inspection of the resultant trajectories showed 2-*O*-caffeoylglucaric acid and rosmarinic acid completely detached from their initial binding site and so were not further considered in this study. On the other hand, the two compounds, caffeoylmalic and chlorogenic acids showed a relatively similar binding mode that was stable throughout the entire simulation. The convergence of the simulation was observed after 25 ns where the root mean square deviation (RMSD) of the backbone atoms of IL-6 levelled off at around 2 Å (Figure 6). For chlorogenic and caffeoylmalic acid, their RMSD showed some fluctuations before stabilizing at about 1.5 and 2 Å, respectively confirming the resultant binding modes of the docked poses**.**

**FIGURE 7:**
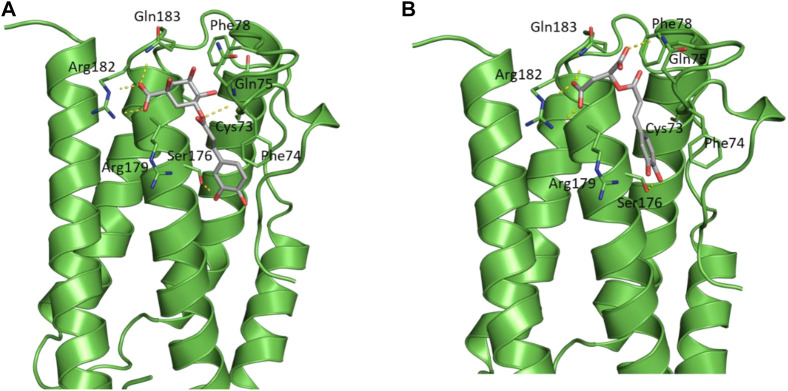
Structural representation of IL-6 (green cartoon) bound to **(A)** chlorogenic acid and **(B)** caffeoylmalic acid (grey sticks).

The docked poses of the two compounds showed their caffeic acid fragments binding in a similar manner **(** Figure 7) with the phenyl ring forming aromatic interactions with the side chain of Phe74 and one of the polar hydroxyl groups forming H-bond with the side chain of Ser176. The malic acid fragment of caffeoylmalic acid showed one of its acidic carboxyl groups forming bidentate ionic interaction with Arg182 and a H-bond with the side chain of Gln183 while the other carboxyl group is involved in H-bond with Gln75. For chlorogenic acid, the acidic carboxyl group of the quinic acid moiety showed strong interactions with Arg182, Arg179 and Gln183 in a similar fashion to the malic acid fragment of caffeoylmalic acid. In accordance, H-bond analysis showed the two compounds maintaining these similar H-bond interactions throughout the simulation (Table 3).

**TABLE 3:** H-bond analysis of the last 50 ns of the MD trajectory of chlorogenic and caffeoylmalic acids bound to IL-6.

HB-acceptor	HB-donor	Donor	Percentage of persistence along the MD trajectory^a^	Average H-bond Distance (Å)	Average H-bond Angle
Chlorogenic acid					
CYS_73@O	LIG_156@H5	LIG_156@O3	45%	2.79	152.9
LIG_156@O5	ARG_179@HE	ARG_179@NE	31%	2.83	156.2
LIG_156@O5	ARG_179@HH22	ARG_179@NH2	30%	2.82	154.5
LIG_156@O6	ARG_179@HH22	ARG_179@NH2	29%	2.81	156.8
LIG_156@O6	ARG_179@HE	ARG_179@NE	25%	2.85	155.7
CYS_73@O	LIG_156@H6	LIG_156@O4	23%	2.74	156.2
SER_176@OG	LIG_156@H1	LIG_156@O8	18%	2.72	165.9
LIG_156@O6	ARG_182@HH21	ARG_182@NH2	16%	2.77	160.3
LIG_156@O5	ARG_182@HE	ARG_182@NE	11%	2.86	165.2
LIG_156@O4	GLN_75@HE21	GLN_75@NE2	11%	2.88	160.6
Caffeoylmalic acid					
SER_176@OG	LIG_156@H3	LIG_156@O6	48%	2.78	160.1
LIG_156@O3	ARG_182@HH21	ARG_182@NH2	29%	2.73	163.1
LIG_156@O7	ARG_179@HH22	ARG_179@NH2	27%	2.80	157.9
LIG_156@O3	ARG_179@HH22	ARG_179@NH2	24%	2.81	159.8
LIG_156@O8	GLN_75@H	GLN_75@N	22%	2.87	161.5
LIG_156@O7	ARG_182@HE	ARG_182@NE	21%	2.81	160.2
LIG_156@O3	ARG_179@HE	ARG_179@NE	16%	2.84	159.5
LIG_156@O4	GLN_75@HE21	GLN_75@NE2	13%	2.85	163.7
LIG_156@O7	GLN_183@HE22	GLN_183@NE2	13%	2.85	152.3

^a^ Only H-bonds that are persistent in more than 10% of the snapshots are included. For each H-bond, column 1 represents the acceptor residue and atom name. Columns 2 and 3 indicate the name of the H-atom and the electronegative atom attached to it on the donor residue, respectively.

Finally, binding free energy was calculated for the two compounds using the MM-GBSA method based on frames sampled every 10 ps from the last 50 ns of the trajectories (Table 4) (Genheden and Ryde, 2015). The binding free energies of chlorogenic and caffeoylmalic acids were −20.9 and −17.1 kcal/mol; respectively, indicating a relatively better binding affinity of chlorogenic acid.

**TABLE 4:** MM-GBSA binding free energies (kcal/mol) for chlorogenic and caffeoylmalic acids bound to IL-6.

Compound	MM-GBSA (kcal/mol)	Std deviation	Std. Error of mean
**Chlorogenic acid**	−20.9	4.7	0.22
**Caffeoylmalic acid**	−17.1	4.8	0.23

#### *Leonotis ocymifolia* (LO) diminishes renal structural changes induced by cisplatin

**FIGURE 8:**
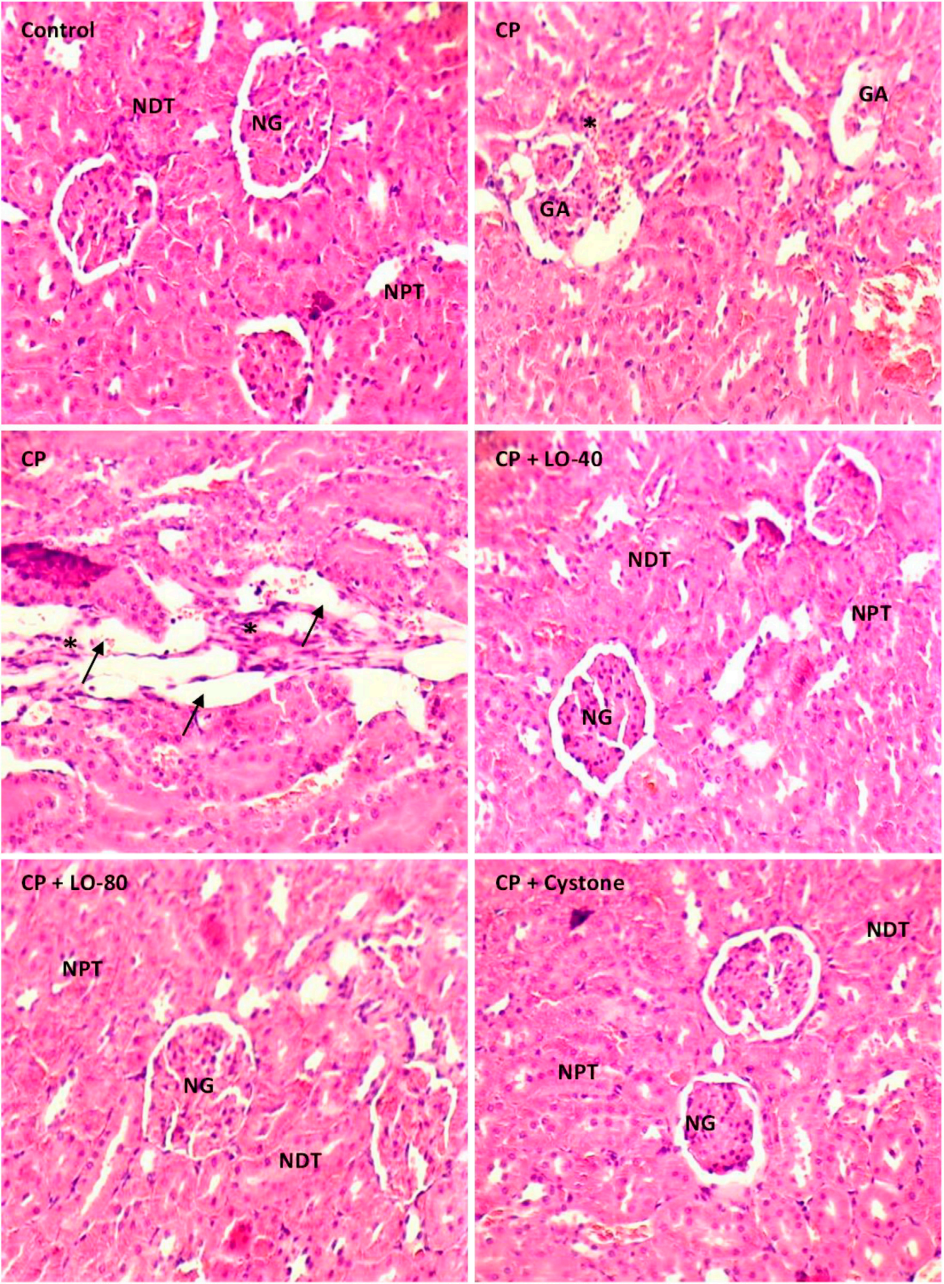
Photomicrographs of the kidney tissue stained with H and E following 30 days pretreatment with *Leonotis ocymifolia* (LO, 40 and 80 mg/kg, PO) and cystone as reference drug (100 mg/kg) followed by single dose cisplatin (CP, 13 mg/kg, IP) (200x, Normal glomeruli (NG), Normal distal tubules (NDT), Normal proximal tubules (NPT). Glomerular atrophy (GA), tubular dilatation (arrow) and infiltration of immune cells (asterix)).

The histological analysis using H and E staining revealed that the control group exhibited normal renal structures (Figure 8). The kidney sections showed intact renal corpuscles, with large number of glomeruli, each of which surrounded by narrow Bowman’s spaces as well as normal distal and proximal convoluted tubules. Renal tissues from cisplatin-treated rats revealed excessive glomerular atrophy with presence of a large void in the capsule space, tubular fibrosis, tubular dilatation, and infiltration of immune cells, compared to that from the control group. Pre-treatment of the rats either with LO (40) or LO (80) significantly diminished the cisplatin-induced alteration in the renal tissue. No significant differences were observed between LO and cystone groups.

#### *Leonotis ocymifolia* (LO) prevents renal DNA fragmentation

**FIGURE 9:**
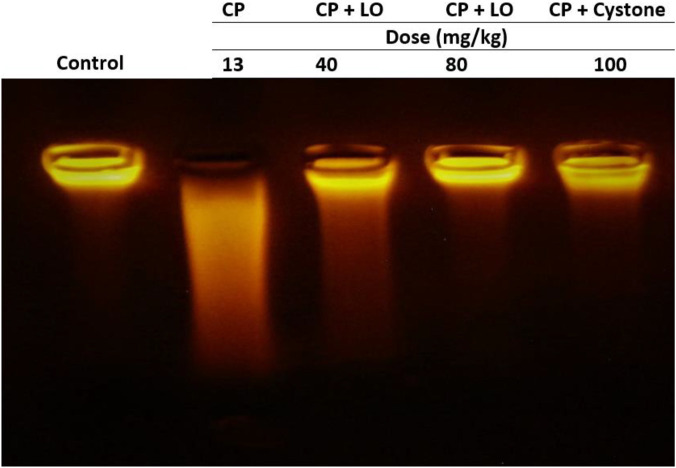
*Leonotis ocymifolia* (LO) protected rat kidney against cisplatin-induced genomic DNA fragmentation. An electrogram of the rat kidneys following 30 days pretreatment with *Leonotis ocymifolia* (LO, 40 and 80 mg/kg, PO) and cystone as reference drug (100 mg/kg) followed by single dose cisplatin (CP, 13 mg/kg, IP) (*n* = 6).

The integrity of DNA recovered from the kidney tissues of various experimental groups underwent several qualitative alterations following cisplatin administration, as shown in Figure 9. DNA electrophoresis on an agarose gel demonstrated that cisplatin caused genotoxicity. A fragmented DNA was observed in cisplatin treated group compared to control group (Figure 9). No tailing of DNA fragments was observed in LO or cystone pretreated rats compared to cisplatin group, indicating antigenotoxicity effect of LO (Figure 9).

## Discussion

Cisplatin received approval for medical use in the eighth decade of 20th century to treat a variety of malignancies (Kerpel‐Fronius, 2006; Cullen et al., 2007; Vokes, 2010; Ismaili et al., 2011). Unfortunately, it has several negative side effects that limit its use, such as neurotoxicity, electrolyte imbalance, hemolytic anemia, nausea and vomiting (Volarevic et al., 2019). However, nephrotoxicity is a more serious concern as it is dose dependent and limits its anticancer effects. The mechanisms of nephrotoxicity are still not well-understood.

Although, creatinine, uric acid and urea tests are screening assays employed to evaluate renal function, creatinine is the most specific marker for renal injury (Siew et al., 2011). Reflecting on previous reports suggesting that urea is formed by the liver when dietary or tissue protein is metabolized, and it is excreted through the kidney (Sun et al., 2022). The administration of cisplatin leads to an upregulation of renal biomarkers, namely, creatinine, uric acid and urea which indicates potential kidney damage. A similar effect was reported earlier, which revealed a significant increase in these parameters (Yassir et al., 2022).

The present study demonstrates the nephroprotective effect of *L. ocymifolia* at different doses in rat model of cisplatin-induced nephrotoxicity. Overall, our results elucidate that pre-treatment with LO exhibits a dose-dependent modulation of cisplatin-induced renal dysfunction by reducing uric acid levels and mitigating serum creatinine levels compared to the control group. These observations strongly support the notion of a potential protective effect on renal function by LO. However, it is noteworthy that the extract and the reference drug, cystone concurrently induces an elevation serum urea level.

The kidney is responsible for eliminating 90% of the metabolized urea, which is a major nitrogenated metabolite originating from protein degradation in the body (Saatkamp et al., 2016). Indeed, the primary means of transporting urea, the metabolic byproduct of amino acids, is through urea transporters (UTs) (Fenton and Knepper, 2007). The rate at which urea is transported across cell membranes is 10–100 times faster when it is transported specifically by UTs. There are two main isoforms of UTs, UT-A (SLC14A2) and UT-B (SLC14A1), that control the concentration of urine in the kidney and perform crucial roles in the local kidney urea cycles (Klein et al., 2011). UT-A1 is expressed in the inner medullary collecting duct principal cells and UT-A2 is found in the thin descending limb of Henle (Nielsen et al., 1996; Wade et al., 2000). It was observed that there was increased glycosylation of UT-A2 and/or UT-A4 proteins in cisplatin toxicity which may affect their function and may contribute to cisplatin induced polyurea (Sands, 1999; Ecelbarger et al., 2001). Changes in the expression or function of these transporters will affect serum level of urea and affect urine osmolarity. One explanation for the increased urea level by the extract and cystone is that they may either potentiate cisplatin effects on urea transporters or they may have direct effects on them resulting to elevated urea level. The precise underlying mechanisms governing the augmented urea levels induced by the plant extract remain elusive and our hypothesis warrants further investigation.

The kidneys play an indispensable role in maintaining sodium and potassium homeostasis, and their diminished functionality gives rise to electrolyte imbalances. About 70% of the sodium reabsorption from the glomerular ultrafiltrate occurs in the proximal tubule cells (Li and Zhuo, 2016). The most significant proximal tubule sodium transporter is the sodium hydrogen exchanger 3 (NHE3), which is found in the apical membrane of the proximal tubule cells (Li et al., 2018). Out of the 20 various cell types in the human kidney, the renal proximal tubule epithelial cell is the major target of renal damage due to its specific roles. Cisplatin is absorbed and accumulated in proximal tubular cells and triggers oxidative stress, inflammation, vascular injury, cell apoptosis, necrosis, and endoplasmic reticulum stress and impaired expression of different transporters (Jing et al., 2022). Cisplatin induced proximal tubular injury leads to decreased sodium reabsorption and hyponatremia. This is confirmed by our findings that revealed a significant reduction in serum sodium concentration and an elevation in potassium levels following the administration of cisplatin. Interestingly, treatment with LO demonstrated a dose-dependent effect, effectively restored the diminished serum sodium level, and partially attenuated the elevated potassium concentration compared to the cisplatin group. The effect of the extract on the serum sodium might be due to a protective effect on proximal tubular cells. The present findings were in contrast with the previous studies showing that cisplatin induced potassium wasting in urine leading to hypokalemia (Yao et al., 2007). The obtained results could be due to reduction in glomerular filtration by cisplatin which could produce damage to the proximal tubule and lead to the elevation of uremic toxins and potassium levels. Previous study showed a decreased in glomerular filtration rate in rats treated with cisplatin at low dose for 10 weeks adding support to the findings of the present work (El-Waseif et al., 2022).

Reactive oxygen species (ROS) are byproducts of metabolism and cellular respiration, including superoxide (O2•) and hydroxyl radicals (OH•), which are extremely reactive and harmful (Powers and Jackson, 2008). However, superoxide dismutase (SOD) generally converts O2• to nonradical H_2_O_2_, which is then degraded by catalase, and glutathione peroxidases (GPXs) (Sies and Jones, 2020). An important factor in the kidney damage brought on by cisplatin is the oxidative stress (Manohar and Leung, 2018). When cisplatin is administered, ROS build up and induce cell damage, including apoptotic cell death (Pei et al., 2022). Cisplatin treatment results in an increase in ROS levels alongside a decrease in the activity of antioxidants, including SOD, which can neutralize ROS (Pei et al., 2022). SOD levels significantly dropped following cisplatin administration (Huang et al., 2020). The current study revealed that the administration of cisplatin elicited an accumulation of various reactive oxygen species (ROS), resulting in depleting the antioxidant system and activating the antioxidant enzymes. As evidenced by our findings, catalase (CAT) and superoxide dismutase (SOD) activities were found to be reduced in rats treated with cisplatin, alongside a decrease in glutathione (GSH) levels when compared to the control groups; this finding aligns with previous reports (Yassir et al., 2022; Anwer et al., 2023). Furthermore, an elevation in MDA levels was observed, indicating an augmentation in lipid peroxidation. The pretreatment with LO effectively reversed the decreased levels of antioxidant enzymes and restored the depleted GSH content. Additionally, it successfully reduced the elevated lipid peroxidation levels closer to the normal range. These results strongly suggest the remarkable antioxidant and free radical scavenging capacities of LO, which effectively shielded the kidney from free radical-induced damage during cisplatin toxicity. The antioxidant properties of LO extract are mainly due to its polyphenolic content.

Various compounds found in plants exhibit distinct antioxidant properties, demonstrating their capability to scavenge and neutralize ROS, thereby mitigating oxidative stress and its associated detrimental effects. Thus, the exploration of these plant-derived compounds as potential therapeutic agents holds great promise for addressing oxidative stress-related disorders and promoting overall health and wellbeing. LC-MS results clearly showed that LO contained various flavonoids and phenolics, including quercetin, ferulic, rosmarinic, protocatechuic, caffeic and chlorogenic acids. Caffeic acid and its derivatives consistently demonstrate potent antioxidant activity. Chlorogenic acid exhibit potent antioxidant activity by increasing superoxide dismutase, catalase, and glutathione and reducing lipid peroxidation in rats (Sato et al., 2011). It is well established that the antioxidant capacity of rosmarinic acid makes this compound a good drug candidate for treatment of oxidative stress-related pathological conditions (Domitrović et al., 2014; Elufioye and Habtemariam, 2019).

During cisplatin nephrotoxicity, inflammation plays a crucial role. It is largely known that inflammation contributes to kidney injury following cisplatin treatment (Tan et al., 2019). It has been proved that oxidative stress causes local inflammation *via* the upregulation of pro-inflammatory cytokines, including IL-6. IL-6 is a characteristic cytokine for maintaining homeostasis (Tanaka et al., 2014). Once IL-6 level is overtly dysregulated and chronically generated, it has detrimental effects and causes acute systemic inflammation. Prior research has demonstrated that IL-6 levels are significantly increased in cisplatin-induced nephrotoxicity (Yassir et al., 2022). These findings confirmed our observation showing increased infiltration of immune cells in renal tissues and elevated renal IL-6 levels following cisplatin administration. Here, the tested extract attenuated cisplatin induced IL-6 elevation. The anti-inflammatory effects of the extract are ascribed primarily to its antioxidant effects. *In silico* results confirmed the anti-inflammatory effect of LO extract by revealing a consistently stable binding mode between IL-6, chlorogenic and caffeoylmalic acids. These specific compounds within the extract are partially accountable for the nephroprotective properties.

It has been demonstrated that reactive oxygen species (ROS) possess the capacity to target and modify various cellular molecules, including lipids, proteins, and DNA, resulting in cellular stress (Sobeh et al., 2018). Furthermore, their involvement in the activation of numerous pathways vital signaling pathways during cisplatin nephrotoxicity has been established (Zhang et al., 2023). In the present study, cisplatin caused renal DNA fragmentation indicating activation of apoptotic cell death. It also suppressed the synthesis of DNA, RNA, and proteins and induced DNA fragmentation either directly by binding to DNA to form covalent platinum DNA adducts and DNA alkylation or indirectly by producing ROS, which is known as one of the pathogenic intermediates causing DNA damage after chemotherapy (Saad et al., 2009). Administration of the tested extract to rats caused a significant reduction of DNA fragmentation. The protective effects of the extract may be mediated by suppression of ROS-induced DNA damage. These results indicated that LO is a worthy candidate to mitigate DNA damage in renal tissues.

Combining histological analysis and biochemical parameters provides a comprehensive assessment. Our investigation revealed that cisplatin induced significant damage to renal tissues. It caused excessive glomerular atrophy with presence of a large void in the capsule space, tubular fibrosis, tubular dilatation, and infiltration of immune cells. These observed alterations are likely attributed to the generation of ROS and subsequent lipid peroxidation and inflammation. By mitigating oxidative stress and suppression of inflammation, LO extract diminished cisplatin-induced alteration in the renal tissue confirming its nephroprotective effects.

## Conclusion

The current study annotated the phytocontents of *L. ocymifolia* aerial parts extract *via* LCMS and highlighted its nephroprotective effects against cisplatin induced kidney injury through inhibition of oxidative stress, inflammation, and DNA fragmentation. Molecular docking and MD simulation studies showed stable binding mode for chlorogenic and caffeoylmalic acids to IL-6 suggesting these compounds to be partially responsible for the observed nephroprotective effect of *L. ocymifolia* extract. Further experiments are required to comprehensively elucidate the involved mechanisms, isolate the phytoconstituents of the extract and explore their activities.

## Data Availability

The original contributions presented in the study are included in the article/Supplementary Material, further inquiries can be directed to the corresponding authors.

## Abbreviations

CAT; Catalase, Cp; Cisplatin, DPPH; 2,2-diphenyl-1-picrylhydrazyl, FRAP; Ferric reducing antioxidant power assay, GPXs; Glutathione peroxidases, GSH; Glutathione, HPLC-PDA-MS/MS; High performance liquid chromatography coupled with UV diode array (PDA) and mass spectrometry detection, IL-6; Interleukin 6, LO; *L. ocymifolia* extract, MDA; Malondialdehyde, ROS; Reactive oxygen species, RPT; Renal proximal tubular, SOD; Superoxide dismutase, TFC; Total flavonoids content, TPC; Total phenolic content, UAE; Ultrasound-assisted extraction, UTs; Urea transporters.
