# Supplementary material for: Leonotis ocymifolia (Burm.f.) Iwarsson aerial parts aqueous extract mitigates cisplatin-induced nephrotoxicity via attenuation of inflammation, and DNA damage
Source: Front Pharmacol. 2023 Aug 1;14:1221486. doi: 10.3389/fphar.2023.1221486 (PMC10428015; doi:10.3389/fphar.2023.1221486)
Supplement: Supplementary file 1 [file Table1.docx]

**SUPLLEMENTARY MATERIAL**

**Table S1.** GOLD scores of the docked poses of extract’s compounds with IL-6.

| **Compound** | **Docking Score** |
| --- | --- |
| 2-*O*-caffeoylglucaric acid | 72.73 |
| Caffeoylmalic acid | 65.8 |
| Rosmarinic acid | 65.08 |
| Chlorogenic acid | 60.96 |
| Malic acid glucoside | 56.84 |
| Galloyl glucose | 54.92 |
| Protocatechuic acid glucoside | 54.16 |
| Coumaric acid glucoside | 49.66 |
